# Micromachined Planar Supercapacitor with Interdigital Buckypaper Electrodes

**DOI:** 10.3390/mi9050242

**Published:** 2018-05-16

**Authors:** Yun-Ting Chen, Cheng-Wen Ma, Chia-Ming Chang, Yao-Joe Yang

**Affiliations:** Department of Mechanical Engineering, National Taiwan University, No. 1, Sec. 4, Roosevelt Rd., Taipei 10617, Taiwan; ytchen@mems.me.ntu.edu.tw (Y.-T.C.); jeson@mems.me.ntu.edu.tw (C.-W.M.); chiaming1129@mems.me.ntu.edu.tw (C.-M.C.)

**Keywords:** carbon nanotube (CNT), micro-supercapacitor (micro-SC), patterned buckypaper, vacuum filtration

## Abstract

In this work, a flexible micro-supercapacitor with interdigital planar buckypaper electrodes is presented. A simple fabrication process involving vacuum filtration method and SU-8 molding techniques is proposed to fabricate in-plane interdigital buckypaper electrodes on a membrane filter substrate. The proposed process exhibits excellent flexibility for future integration of the micro-supercapacitors (micro-SC) with other electronic components. The device’s maximum specific capacitance measured using cyclic voltammetry was 107.27 mF/cm^2^ at a scan rate of 20 mV/s. The electrochemical stability was investigated by measuring the performance of charge-discharge at different discharge rates. Devices with different buckypaper electrode thicknesses were also fabricated and measured. The specific capacitance of the proposed device increased linearly with the buckypaper electrode thickness. The measured leakage current was approximately 9.95 µA after 3600 s. The device exhibited high cycle stability, with 96.59% specific capacitance retention after 1000 cycles. A Nyquist plot of the micro-SC was also obtained by measuring the impedances with frequencies from 1 Hz to 50 kHz; it indicated that the equivalent series resistance value was approximately 18 Ω.

## 1. Introduction

Supercapacitors (SCs) are energy storage devices that provide a higher density of energy than conventional dielectric capacitors, and higher density of power than batteries. SCs are frequently employed to power portable electronic devices because of their efficient charging/discharging performance and capability to reliably operate for millions of cycles. In the past decade, SCs with carbon nanotubes (CNTs) electrodes have drawn global attention because of the unique properties of CNTs, such as high specific surface area, electrical conductivity, and chemical stability [1,2,3,4]; these qualities make CNTs high-quality electrode materials for SCs [5,6,7,8].

Thin films formed with aggregates of CNTs are frequently employed as both current collectors and active materials for flexible SCs [9]. Various techniques for realizing CNT films have been reported. Najafabadi et al. proposed high-power SC electrodes with composites of carbon nanohorns and CNTs [10]; the meso-macro pore structure engineered by employing single-walled CNTs as scaffolding for single-walled carbon nanohorns improved the electrode’s power density. Do et al. proposed a method of vanadium oxide deposition on multi-walled CNT buckypaper that served as SC electrodes [11]. A novel supercritical fluid process was used for the deposition of vanadium oxide onto the buckypapers. In [12], a new technique of synthesizing CNT and nanofiber ensembles using a template method was proposed. Synthesis of carbon by using chemical vapor deposition within the pores of an alumina template membrane was employed. The fabricated carbon nanofiber and nanotube ensembles can be used as electrodes in lightweight SCs. In [13], a novel ternary composite paper composed of reduced graphene sheet (GR)-patched CNT/MnO_2_ was proposed. The proposed composite film can potentially be employed as an electrode for flexible high-performance energy storage devices. Meng et al. proposed an innovative method to prepare buckypaper/polyaniline (BP/PANI) composites, which are relatively thin and exhibit exceptional flexibility compared with conventional brittle CNT/PANI composites [14]; the measured results also revealed excellent electrochemical properties. In [15], a hybrid structure formed using a core-shell structural nanowire network was proposed as the electrode material for SCs. In the proposed hybrid structure, which comprises binder-free carbon nanomaterial, buckypaper films were employed as conductive scaffolds, and CNTs in the buckypaper film were coated with a porous active carbon layer, which served as an active component for enhancing capacitance.

In general, the electrode structures of SCs can be classified into three types: interdigital [16,17,18], sandwich [19,20], and roll [21,22]. In-plane interdigital design of electrodes possesses some advantages over the other two designs. Because the electrodes are in the same plane, micro-SCs can easily be integrated with other electronic components. In addition, it is possible to accurately reduce the gaps between the interdigital electrodes, which in turn decreases the ion transport resistance for SCs [23]. During the past decade, different types of SCs with interdigital electrodes have been reported. Shen et al. introduced a 3D high-aspect-ratio micro-SC that was fabricated by using deep etching techniques. The proposed device exhibited high capacitance and power per unit area [24]. Pech et al. presented a micrometer-sized SC with ultrahigh power based on onion-like carbon. High power density was achieved by electrophoretic deposition of nanostructured carbon onions onto interdigital Au current collectors [25]. In [26], a method for fabricating interdigital-patterned electrodes for micro-SCs was reported. The device employed reduced graphene oxide and CNT composites as the electrode materials. The fabrication process consisted of electrostatic spray deposition combined with photolithography lift-off methods. In [27], high-performance micro-SCs based on PANI nanofibers and graphene quantum dots (GQDs) were presented. The proposed asymmetric device employed GQDs as the negative active material and PANI nanofibers as the positive active material. In [28], flexible micro-SCs were fabricated by laser carbonizing polyimide sheets. Flexible micro-SCs can be fabricated without using photolithographic-patterned porous carbon and metal layers. Lee et al. reported planar-type flexible micro-SC arrays using Au electrodes coated with a functionalized multiwalled CNT (MWCNT) film and an MWCNT-COOH/MnO_x_ composite layer [29]. The porous 3D network electrode structures in the SCs achieved high capacitance and energy density because of effective diffusion of the electrolytes and fast electronic and ionic conduction.

This study proposes an in-plane micro-SC with buckypaper-based interdigital electrodes. Polyvinyl alcohol-potassium hydroxide (PVA-KOH) was used as a gel electrolyte. The fabrication process employed a vacuum filtration method [30] as well as lithography techniques to produce high-aspect-ratio interdigital electrodes. The proposed micro-SCs are flexible and lightweight, and can be easily fabricated. In addition, the performance of the proposed micro-SC was evaluated by conducting various measurements such as cyclic voltammetry (CV), galvanostatic charge-discharge, and cycle stability experiments. The remainder of this paper is organized as follows: The design and principles are described in Section 2; the fabrication is described in Section 3; the measurement results and discussion are provided in Section 4; and finally, Section 5 presents the conclusion.

## 2. Device Design and Operational Principles 

**Figure 1:**
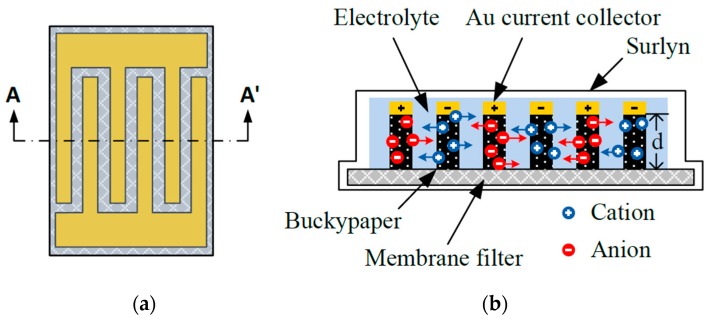
Schematic diagrams of the proposed micro-supercapacitors (micro-SC): (**a**) top-view; and (**b**) cross-sectional view of A-A’.

The schematic of the proposed micro-SC is shown in Figure 1. Figure 1a shows the top view of the proposed device, and Figure 1b shows a cross-sectional view. The proposed device consists of a pair of interdigital buckypaper electrodes fabricated with a typical photolithography process and vacuum filtration technique on a nylon membrane filter (MS^®^ nylon membrane filter, Membrane Solutions Corporation, Westborough, MA, USA) with pore size of 0.8 μm. An Au film (Gold Target, Electron Microscopy Sciences Corporation, Hatfield, PA, USA) of 200 nm was deposited on the top surface of the patterned buckypaper and served as the current collector. Also, the trenches between the electrodes were filled with gel electrolyte.

As shown in Figure 1b, during the micro-SC’s charging process, electrons moved from the positive to the negative electrode via external power sources. Additionally, positive and negative ions in the electrolyte separated and moved to the electrode surfaces, resulting in the formation of electric double layers [31]. The device stored energy because ions of opposite charge accumulated on the double layers of electrochemically stable electrodes with high specific surface area. This study used patterned interdigital buckypaper to serve as the electrode material, which provides high electrochemical stability during the charge-discharge process. Moreover, the high surface-to-volume ratio of the porous buckypaper electrodes caused the micro-SC to have high energy and power density [25]. Also, using a filtration paper as the substrate allowed the proposed in-plane device to be flexible and relatively thin, thus easily integrated with wearable devices.

Notably, the capacity of the proposed in-plane interdigital micro-SC can be increased by increasing the thickness of the buckypaper electrodes; its charge-discharge rates are barely affected because there is almost no increase in the ion migration distance. For SCs with planar sandwiched structures, however, as the thickness of the buckypaper increases, the ion migration distance also increases, which in turn deteriorates the charge-discharge performance [25,32].

## 3. Fabrication

**Figure 2:**
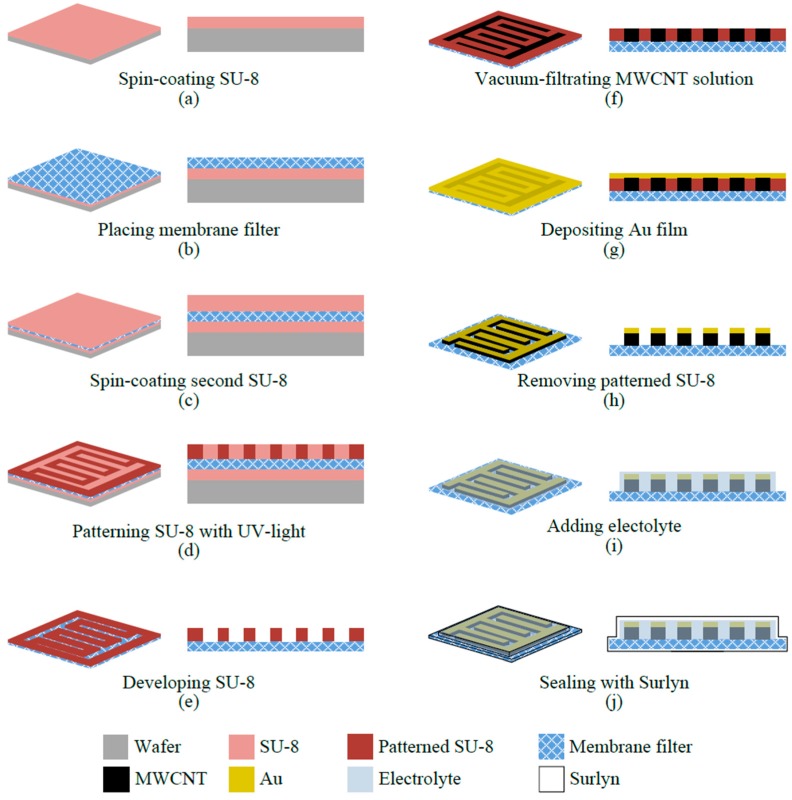
The fabrication process of the supercapacitor with interdigital patterned buckypaper electrodes: (**a**) spin-coating first SU-8 layer; (**b**) placing nylon membrane filter on the top of first SU-8 layer; (**c**) spin-coating second SU-8 layer on nylon membrane filter; (**d**) patterning SU-8 using a standard photolithography process; (**e**) developing SU-8; (**f**) vacuum filtrating MWCNT solution with SU-8 patterned nylon membrane filter; (**g**) depositing Au film on the surface of buckypaper; (**h**) removing SU-8 frame; (**i**) adding electrolyte to electrodes; (**j**) sealing device with polymer films.

The fabrication process of the proposed micro-SC is shown in Figure 2. First, a layer of 170-μm SU-8 thick-film photoresist (SU-8 2050, MicroChem Corporation, Westborough, MA, USA) was spin-coated (20 s at 500 rpm and 50 s at 1000 rpm) onto a silicon handling wafer, as shown in Figure 2a. The model of the spin-coater was SP-01 (APISC Corporation, Taoyuan, Taiwan). Figure 2b shows how a nylon membrane filter, which served as the micro-SC’s flexible substrate, was then placed on the top of the SU-8 photoresist. Then, a second layer of 100-μm SU-8 photoresist was spin-coated (20 s at 500 rpm and 50 s at 1400 rpm) on top of the membrane filter (Figure 2c). The SU-8 layer was patterned (Figure 2d) and developed using a standard photolithography process, forming an SU-8 mold for the interdigital electrodes pair (Figure 2e). Then, CNT solution dispersed with MWCNTs (0.01 wt % concentration) was filtrated through the membrane filter with the SU-8 mold by using the vacuum filtration technique [33]; MWCNTs filled into the SU-8 mold, as shown in Figure 2f. The solution dispersed with MWCNTs was subjected to ultrasonic agitation for 120 min to reduce the CNTs’ tendency to bundle. After vacuum filtration, the MWCNT film was kept at room temperature for 120 min to evaporate residual solvent thoroughly. Then, a 200-nm Au layer, which served as the current collector, was deposited on top of the MWCNT film (Figure 2g). After removing the SU-8 mold with solvent stripper (Remover PG, MicroChem Corporation, Westborough, MA, USA) [24], the patterned interdigital buckypaper electrodes were fabricated (Figure 2h). The removal process was facilitated by the nylon membrane filter’s permeability since the solvent stripper solution easily penetrated the membrane and reached the contact interface between the SU-8 structure and the membrane.

Before packaging the device, PVA-KOH gel electrolyte was added to the buckypaper electrodes using a syringe. Then, the trenches of interdigital porous buckypaper electrodes were filled and soaked with gel electrolyte (Figure 2i). The electrolyte was synthesized by mixing 2.8 g of potassium hydroxide (KOH) and 5 g of polyvinyl alcohol (PVA) with 50 mL of deionized water at 85 °C. Then, the mixture was stirred for 120 min until the solution became clear. Finally, the device was sealed by polymer films (Surlyn^®^, DuPont Corp, Wilmington, DE, USA) (Figure 2j). 

**Figure 3:**
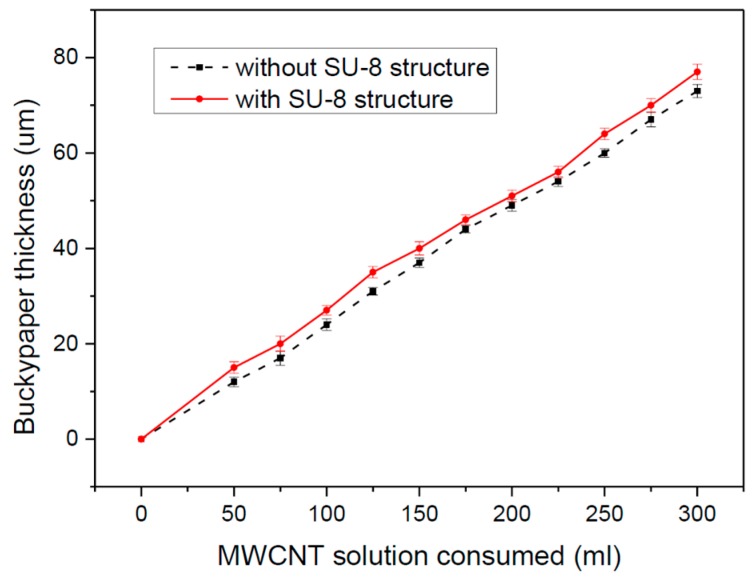
Thickness of buckypaper vs. consumption of MWCNT solution.

The measured relationships between the thickness of the fabricated buckypaper and the consumption of MWCNT solution is shown in Figure 3. The relationships are quite linear. This figure also compares the results of SCs fabricated using filter membranes patterned with SU-8 structures with those using filter membranes without SU-8 structures. The two curves are almost identical, which indicates the SU-8 structure does not affect the filtration’s efficiency. Note that the thickness of the SU-8 frame should be at least 20 μm greater than the buckypaper thickness to ensure successful SU-8 removal after Au film deposition.

**Figure 4:**
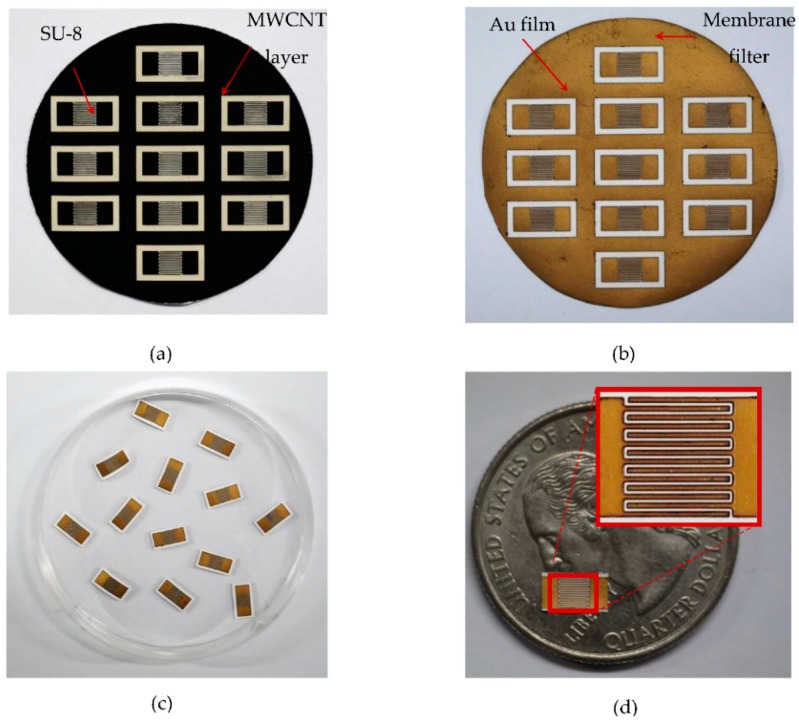
(**a**) The deposited MWCNT film (buckypaper) on the membrane substrate with SU-8 frame after vacuum filtration; (**b**) The micro-SC after depositing the Au layer and removing the SU-8 frame; (**c**,**d**) Fabricated micro-SCs before packaging.

Figure 4a shows the fabricated MWCNT film (buckypaper) on the membrane substrate with an SU-8 frame after vacuum filtration. Figure 4b shows the micro-SC with an Au layer on top of the buckypaper after removing the SU-8 frame with solvent stripper. The fabricated patterned buckypaper electrodes are shown in Figure 4c,d. The size of each micro-SC without contact pads was approximately 3 mm × 3 mm. Each interdigital electrode had seven fingers. The length and width of each finger was 2.73 mm and 80 μm, respectively. The gap between fingers was 100 μm.

**Figure 5:**
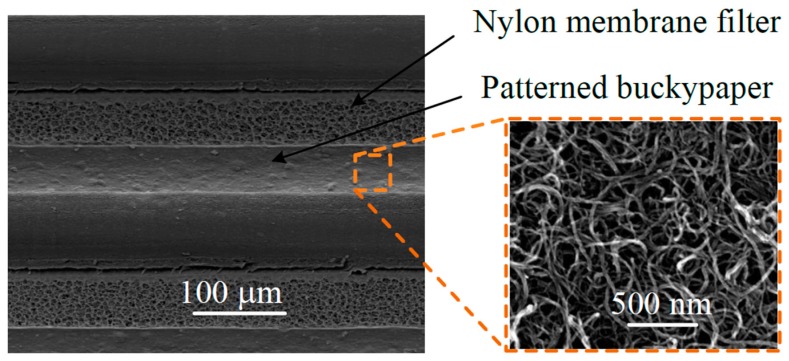
SEM images of the micro-SC.

Figure 5 shows the scanning electron microscopy (SEM) images of the micro-SC (VEGA 3 SBH, TESCAN Corporation, Brno, The Czech Republic). The inset in the figure is an SEM image of higher magnification of the top surface of the patterned buckypaper. The tailored pore structures of the buckypaper, as seen under SEM, are of excellent ion accessibility. 

## 4. Measurement and Discussion

**Figure 6:**
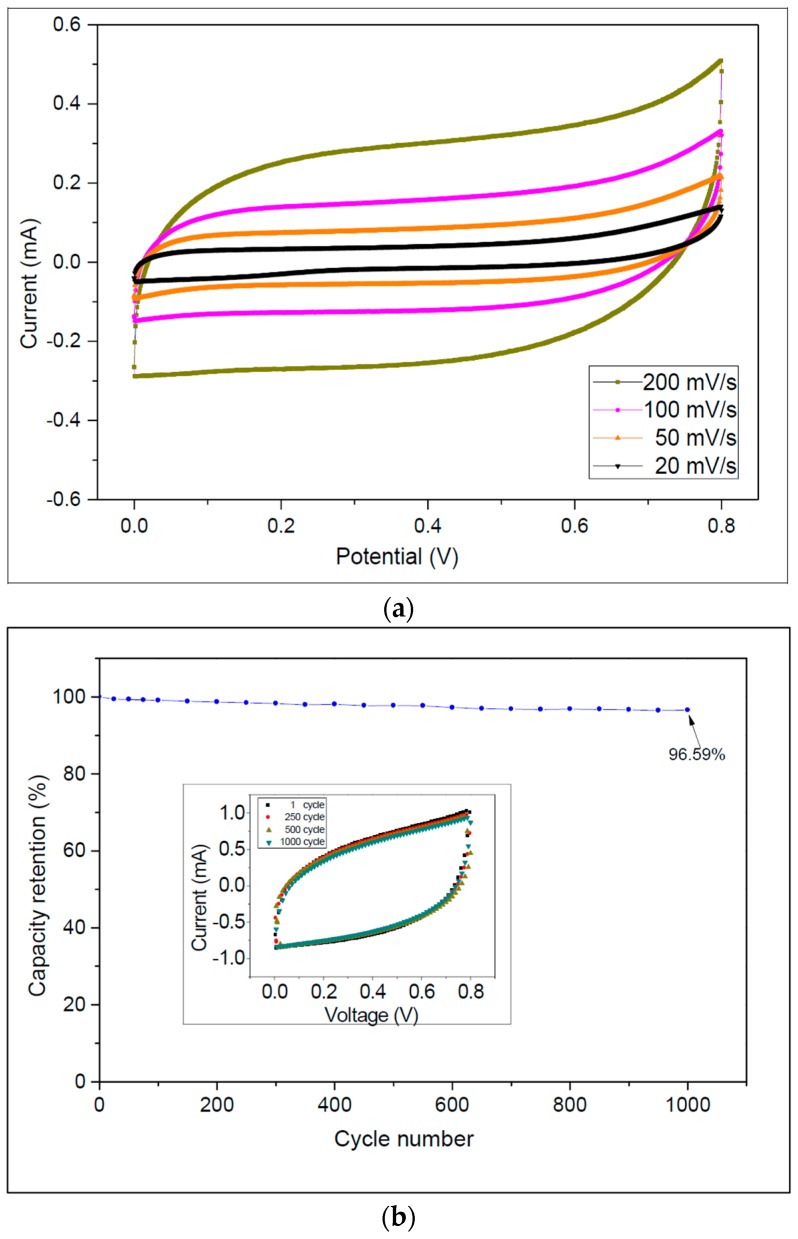
(**a**) Cyclic voltammetry (CV) curve at scanning rates from 20 to 200 mV/s; (**b**) Cycle stability of the micro-SC at a scan rate of 1 V/s. The inset is the CV curves of the 1st, 250th, 750th, and 1000th cycles.

The CV curves of the capacitor at different scanning rates, which were measured using an electrochemical station (CHI 627D, CH Instruments, Austin, TX, USA), are shown in Figure 6a. The proposed device exhibited typical capacitive behavior with quasi-rectangular CV curves. In addition, specific capacitances can be evaluated by using Equation (1) [33]:

(1) $$C_{S}=\frac{AREA_{CV}}{s\cdot A\cdot \Delta V},$$

where *AREA_CV_* is the integral area of a CV curve obtained by integrating the forward and backward sweeps in the cyclic voltammogram, *A* is the total active area of the buckypaper electrodes, *s* is the potential scanning rate, and Δ*V* is the range of the potential sweep. The calculated specific capacitances at different scan rates are listed in Table 1.

Obviously, at very low scan rates, the capacitance values are higher because the ions have sufficient time to penetrate and reside in all the available pores on electrodes, thereby forming electric double layers, which are essential to yield larger capacitance. Using Equation (1), a maximum specific capacitance of 107.27 mF/cm^2^ was obtained at a scan rate of 20 mV/s. 

Devices with different electrode thicknesses were also fabricated and measured. Table 2 shows the results for each electrode configuration at a scan rate of 20 mV/s. The results indicate that the specific capacitance of the devices increased linearly with the thickness of the buckypaper electrodes. Table 3 shows the comparison of specific capacitances among SC with interdigitated electrodes published in recent works. The proposed micro-SC of this work exhibits excellent performance.

Figure 6b shows the capacitance retention ratio versus the number of repeating CV cycles at a scan rate of 1 V/s. The specific capacitance retained 96.59% of its initial value after 1000 cycles. The figure inset shows the CV curves of the 1st, 250th, 750th, and 1000th cycles. These results indicate that the proposed micro-SC has satisfactory cycle stability. 

**Figure 7:**
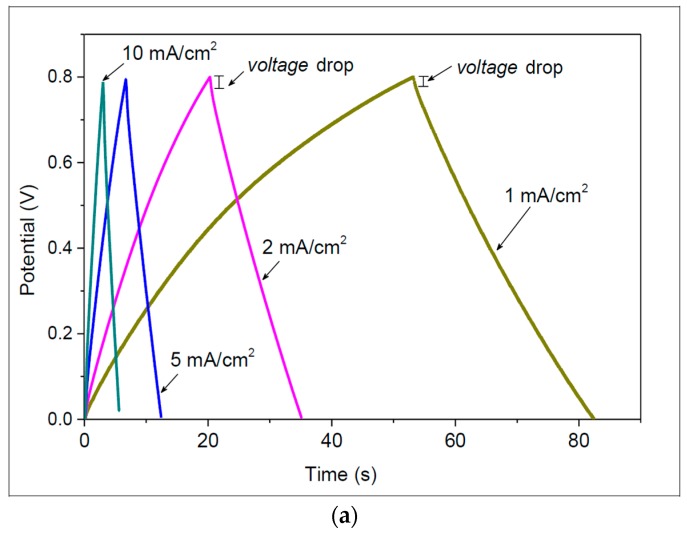

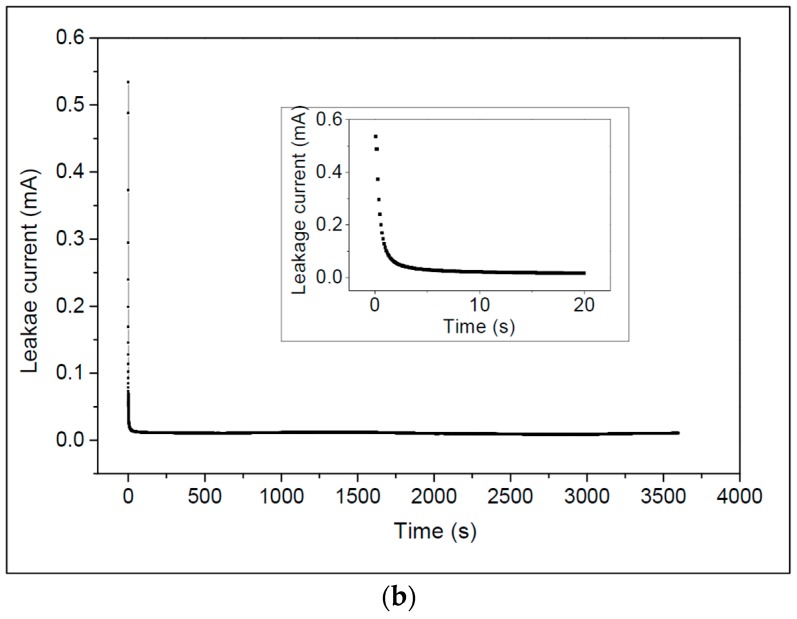
(**a**) Galvanostatic charge-discharge curves at various current densities. (**b**) Leakage current vs. time as the device was charged to 0.8 V and kept at 0.8 V for 3600 s. The charging current was 2 mA.

The proposed device was also tested by galvanostatic charge-discharge cycling at various current densities, as shown in Figure 7a. The corresponding current densities of these curves are 1, 2, 5, and 10 mA/cm^2^. The linear galvanostatic discharge shows that the proposed SC exhibits excellent capacitive behaviors. Note that a small voltage drop at the start of the discharge curve for each galvanostatic charge-discharge curve indicates the existence of internal resistance.

The galvanostatic charge-discharge curves can also be used to evaluate the specific capacitance of the SC by the following equation:

(2) $$C_{d}=\frac{i\cdot \Delta t}{A\cdot \Delta V},$$

where *i* is the discharge current, Δ*V* is the potential drop during discharge, and *A* is the total active area of electrodes. The calculated maximum specific capacitance using Equation (2) was 76.5 mF/cm^2^ at a constant current of 1 mA/cm^2^. Table 4 shows the calculated specific capacitance for each current density. 

Figure 7b shows the leakage current curves of the device, which was charged at 2 mA from 0.0 to 0.8 V, and then maintained at 0.8 V for 3600 s. At the onset of the charging, the leakage current dropped significantly (from 0.534 mA to 19.8 µA after 10 s). The leakage current then decreased gradually and reached a steady value of approximately 9.95 µA after 3600 s. 

**Figure 8:**
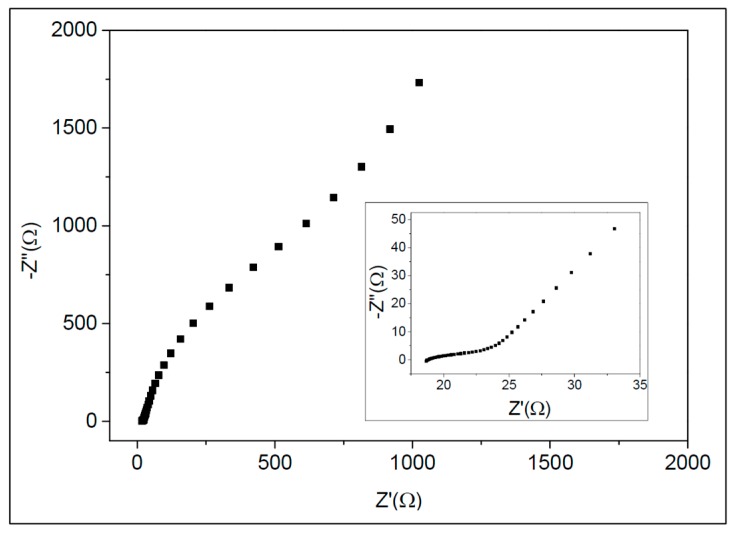
Nyquist plot of the micro-SC. The impedances were measured at a bias of 0.1 V, at frequencies from 20 Hz to 10 kHz.

A Nyquist plot of the micro-SC is shown in Figure 8. The impedances were measured with frequencies from 1 Hz to 50 kHz. At high frequencies, the micro-SC behaved as a resistor, whereas at low frequencies, it behaved as a capacitor. The measured resistance is a combination of various contributions, including the electronic resistance of the patterned buckypaper, the contact resistance between the buckypaper and current collector, and the electrolytic resistance of the buckypaper’s porous structure. The equivalent series resistance value was approximately 18 Ω.

## 5. Conclusions

This paper presents a flexible micro-supercapacitor with interdigital buckypaper electrodes realized by a fabrication process including vacuum filtration and lithography techniques. An SU-8 photoresist layer, which served as the filtration mask, was deposited on a nylon membrane filter and patterned as the mold for an interdigital buckypaper electrode. The device’s electrochemical stability was confirmed by the CV and charge-discharge experiments. The measured maximum specific capacitance was 107.27 mF/cm^2^ at a scan rate of 20 mV/s. Devices with different electrode thicknesses were also fabricated and measured to study the relationship between specific capacitance and buckypaper electrode thickness. In addition, a Nyquist plot of the micro-SC obtained by measuring the impedances showed a resistance value of approximately 18 Ω at high frequency. A small leakage current of 9.95 µA was observed at 3600 s after charging to 0.8 V. The specific capacitance of the device retained 96.59% of its initial value after 1000 cycles.

## Figures and Tables

**Table 1:** Specific capacitances at different scan rates.

Scan Rate (mV/s)	200	100	50	20
*C_s_* (mF/cm^2^)	85.03	89.83	94.19	107.3

**Table 2:** Specific capacitances of different buckypaper thicknesses at 20 mV/s scan rate.

Thickness (µm)	75	60	48	37
*C_s_* (mF/cm^2^)	107.27	82.25	61.62	40.12

**Table 3:** Specific capacitances at different scan rates. PVA-KOH = Polyvinyl alcohol-potassium hydroxide.

Reference	Specific Capacitance (mF/cm^2^)	Electrolyte Material	Fabrication Method	Electrode Material
This work	107.3	PVA-KOH	Vacuum filtration	MWCNT
[34]	2.32	PVA-H_2_SO_4_	Direct laser writing	Graphene
[35]	0.6	Ionogel	Pyrolysis	Photoresistderived porous carbon
[26]	2.8	KCL	Electrostatic spray deposition	Graphene and CNT
[36]	0.53	EMIMBF_4_	Electrophoretic deposition	Graphene quantum dots

**Table 4:** Specific capacitances at various discharge current densities.

Current Density (mA/cm^2^)	10	5	2	1
Δ*t* (s)	2.6	5.7	14.8	30.6
*C_d_* (mF/cm^2^)	65.0	71.2	74.0	76.5

## References

[1] Zhang J., Jiang J., Li H., Zhao X.S. (2011). A High-performance Asymmetric Supercapacitor Fabricated with Graphene-Based Electrodes. Energy Environ. Sci..

[2] Ramadoss A., Kim S.J. (2013). Improved Activity of a Graphene–TiO_2_ Hybrid Electrode in an Electrochemical Supercapacitor. Carbon.

[3] Chen L.F., Huang Z.H., Liang H.W., Yao W.T., Yu Z.Y., Yu S.H. (2013). Flexible All-Solid-State High-Power Supercapacitor Fabricated with Nitrogen-Doped Carbon Nanofiber Electrode Material Derived from Bacterial Cellulose. Energy Environ. Sci..

[4] Koo Y., Shanov V.N., Yun Y. (2016). Carbon nanotube paper-based electroanalytical devices. Micromachines.

[5] Kaempgen M., Ma J., Gruner G., Wee G., Mhaisalkar S.G. (2007). Bifunctional Carbon Nanotube Networks for Supercapacitors. Appl. Phys. Lett..

[6] Niu Z., Zhou W., Chen J., Feng G., Li H., Hu Y., Ma W., Dong H., Li J., Xie S. (2013). A Repeated Halving Approach to Fabricate Ultrathin Single-Walled Carbon Nanotube Films for Transparent Supercapacitors. Small.

[7] Wang Y., Wei H., Lu Y., Wei S., Wujcik E.K., Guo Z. (2016). Multifunctional Carbon Nanostructures for Advanced Energy Storage Applications. Nanomaterials.

[8] Choi H., Yoon H. (2016). Nanostructured Electrode Materials for Electrochemical Capacitor Applications. Nanomaterials.

[9] Chen H., Zeng S., Chen M., Zhang Y., Li Q. (2015). Fabrication and Functionalization of Carbon Nanotube Films for High-performance Flexible Supercapacitors. Carbon.

[10] Izadi-Najafabadi A., Yamada T., Futaba D.N., Yudasaka M., Takagi H., Hatori H., Iijima S., Hata K. (2011). High-Power Supercapacitor Electrodes from Single-Walled Carbon Nanohorn/Nanotube Composite. ACS Nano.

[11] Do Q.H., Zeng C., Zhang C., Wang B., Zheng J. (2011). Supercritical Fluid Deposition of Vanadium Oxide on Multi-Walled Carbon Nanotube Buckypaper for Supercapacitor Electrode Application. Nanotechnology.

[12] Che G., Lakshmi B.B., Martin C.R., Fisher E.R., Ruoff R.S. (1998). Chemical Vapor Deposition Based Synthesis of Carbon Nanotubes and Nanofibers Using a Template Method. Chem. Mater..

[13] Jin Y., Chen H., Chen M., Liu N., Li Q. (2013). Graphene-Patched CNT/MnO_2_ Nanocomposite Papers for the Electrode of High-Performance Flexible Asymmetric Supercapacitors. ACS Appl. Mater. Interfaces.

[14] Meng C., Liu C., Fan S. (2009). Flexible Carbon Nanotube/Polyaniline Paper-Like Films and Their Enhanced Electrochemical Properties. Electrochem. Commun..

[15] Chen H., Di J., Jin Y., Chen M., Tian J., Li Q. (2013). Active Carbon Wrapped Carbon Nanotube Buckypaper for the Electrode of Electrochemical Supercapacitors. J. Power Sources.

[16] Chmiola J., Largeot C., Taberna P.-L., Simon P., Gogotsi Y. (2010). Monolithic Carbide-Derived Carbon Films for Micro-Supercapacitors. Science.

[17] Zhu P., Cai T. (2015). Selection and preparation of the membrane electrode material for micro-Supercapacitor. Sens. Actuators B Chem..

[18] Sette D., Girod S., Leturcq R., Glinsek S., Defay E. (2017). Transparent Ferroelectric Capacitors on Glass. Micromachines.

[19] Meng C., Liu C., Chen L., Hu C., Fan S. (2010). Highly Flexible and All-Solid-State Paperlike Polymer Supercapacitors. Nano Lett..

[20] Wang D.-W., Li F., Zhao J., Ren W., Chen Z.-G., Tan J., Wu Z.-S., Gentle I., Lu G.Q., Cheng H.-M. (2009). Fabrication of Graphene/Polyaniline Composite Paper via In Situ Anodic Electropolymerization for High-Performance Flexible Electrode. ACS Nano.

[21] Ji H., Mei Y., Schmidt O.G. (2010). Swiss Roll Nanomembranes with Controlled Proton Diffusion as Redox Micro-supercapacitors. Chem. Commun..

[22] Sharma R., Bufon C.C.B., Grimm D., Sommer R., Wollatz A., Schadewald J., Thurmer D.J., Siles P.F., Bauer M., Schmidt O.G. (2014). Large-Area Rolled-Up Nanomembrane Capacitor Arrays for Electrostatic Energy Storage. Adv. Energy Mater..

[23] Beidaghi M., Gogotsi Y. (2014). Capacitive Energy Storage in Micro-Scale Devices: Recent Advances in Design and Fabrication of Microsupercapacitors. Energy Environ. Sci..

[24] Shen C., Wang X., Zhang W., Kang F. (2011). A High-Performance Three-Dimensional Micro Supercapacitor Based on Self-Supporting Composite Materials. J. Power Sources.

[25] Pech D., Brunet M., Durou H., Huang P., Mochalin V., Gogotsi Y., Taberna P.-L., Simon P. (2010). Ultrahigh-Power Micrometre-Sized Supercapacitors Based on Onion-Like Carbon. Nat. Nano.

[26] Beidaghi M., Wang C. (2012). Micro-Supercapacitors Based on Interdigital Electrodes of Reduced Graphene Oxide and Carbon Nanotube Composites with Ultrahigh Power Handling Performance. Adv. Funct. Mater..

[27] Liu W., Yan X., Chen J., Feng Y., Xue Q. (2013). Novel and High-Performance Asymmetric Micro-Supercapacitors Based on Graphene Quantum Dots and Polyaniline Nanofibers. Nanoscale.

[28] In J.B., Hsia B., Yoo J.-H., Hyun S., Carraro C., Maboudian R., Grigoropoulos C.P. (2015). Facile Fabrication of Flexible All Solid-State Micro-Supercapacitor by Direct Laser Writing of Porous Carbon in Polyimide. Carbon.

[29] Lee G., Kim D., Yun J., Ko Y., Cho J., Ha J.S. (2014). High-performance All-Solid-State Flexible Micro-Supercapacitor Arrays with Layer-by-Layer Assembled MWNT/MnOx Nanocomposite Electrodes. Nanoscale.

[30] Hennrich F., Lebedkin S., Malik S., Tracy J., Barczewski M., Rösner H., Kappes M. (2002). Preparation, Characterization and Applications of Free-Standing Single Walled Carbon Nanotube Thin Films. Phys. Chem. Chem. Phys..

[31] Zheng J.P., Huang J., Jow T.R. (1997). The Limitations of Energy Density for Electrochemical Capacitors. J. Electrochem. Soc..

[32] Ma C.W., Huang P.C., Yang Y.J. A Paper-like Micro-Supercapacitor with Patterned Buckypaper Electrodes Using a Novel Vacuum Filtration Technique. Proceedings of the 28th IEEE International Conference on Micro Electro Mechanical Systems (MEMS).

[33] Sears K., Dumée L., Schütz J., She M., Huynh C., Hawkins S., Duke M., Gray S. (2010). Recent Developments in Carbon Nanotube Membranes for Water Purification and Gas Separation. Materials.

[34] El-Kady M.F., Kaner R.B. (2013). Scalable Fabrication of High-power Graphene Micro-Supercapacitors for Flexible and On-Chip Energy Storage. Nat. Commun..

[35] Wang S., Hsia B., Carraro C., Maboudian R. (2014). High-Performance All Solid-State Micro-Supercapacitor Based on Patterned Photoresist-Derived Porous Carbon Electrodes and an Ionogel Electrolyte. J. Mater. Chem. A.

[36] Liu W.-W., Feng Y.-Q., Yan X.-B., Chen J.-T., Xue Q.-J. (2013). Superior Micro-Supercapacitors Based on Graphene Quantum Dots. Adv. Funct. Mater..

